# Anti-Tumorigenic and Anti-Metastatic Activity of the Sponge-Derived Marine Drugs Aeroplysinin-1 and Isofistularin-3 against Pheochromocytoma In Vitro

**DOI:** 10.3390/md16050172

**Published:** 2018-05-20

**Authors:** Nicole Bechmann, Hermann Ehrlich, Graeme Eisenhofer, Andre Ehrlich, Stephan Meschke, Christian G. Ziegler, Stefan R. Bornstein

**Affiliations:** 1 Institute of Clinical Chemistry and Laboratory Medicine, University Hospital Carl Gustav Carus, Technical University Dresden, Fetscherstrasse 74, 01307 Dresden, Germany; Graeme.eisenhofer@uniklinikum-dresden.de; 2 Institute of Experimental Physics, TU Bergakademie Freiberg, Leipziger 23, 09599 Freiberg, Germany; Hermann.Ehrlich@physik.tu-freiberg.de; 3 Department of Medicine III, University Hospital Carl Gustav Carus, Technical University Dresden, Fetscherstrasse 74, 01307 Dresden, Germany; Christian.ziegler@uniklinikum-dresden.de (C.G.Z.); Stefan.bornstein@uniklinikum-dresden.de (S.R.B.); 4 BromMarin GmbH, Wernerstraße 1, 09599 Freiberg, Germany; Andre.ehrlich@brommarin.de (A.E.); Stephan.meschke@brommarin.de (S.M.); 5 Center for Regenerative Therapies Dresden, Technical University Dresden, Fetscherstrasse 105, 01307 Dresden, Germany

**Keywords:** marine sponges, Aeroplysinin, Isofistularin, pheochromocytoma and paraganglioma, metastasis, cancer progression, cell adhesion molecules, integrin β1, hypoxia

## Abstract

Over 10% of pheochromocytoma and paraganglioma (PPGL) patients have malignant disease at their first presentation in the clinic. Development of malignancy and the underlying molecular pathways in PPGLs are poorly understood and efficient treatment strategies are missing. Marine sponges provide a natural source of promising anti-tumorigenic and anti-metastatic agents. We evaluate the anti-tumorigenic and anti-metastatic potential of Aeroplysinin-1 and Isofistularin-3, two secondary metabolites isolated from the marine sponge *Aplysina aerophoba*, on pheochromocytoma cells. Aeroplysinin-1 diminished the number of proliferating cells and reduced spheroid growth significantly. Beside these anti-tumorigenic activity, Aeroplysinin-1 decreased the migration ability of the cells significantly (*p* = 0.01), whereas, the invasion capacity was not affected. Aeroplysinin-1 diminished the high adhesion capacity of the MTT cells to collagen (*p* < 0.001) and, furthermore, reduced the ability to form spheroids significantly. Western Blot and qRT-PCR analysis showed a downregulation of integrin β1 that might explain the lower adhesion and migration capacity after Aeroplysinin-1 treatment. Isofistularin-3 showed only a negligible influence on proliferative and pro-metastatic cell properties. These in vitro investigations show promise for the application of the sponge-derived marine drug, Aeroplysinin-1 as anti-tumorigenic and anti-metastatic agent against PPGLs for the first time.

## 1. Introduction

Pheochromocytomas and extra-adrenal paragangliomas are rare neural crest-derived tumors with a highly heterogeneous genetic background associated with variable diseases aggressiveness. Pheochromocytoma generally occurs as a benign tumor, but over 10% are malignant at the first presentation or at recurrence usually with metastases at lymph node, bone, liver, and/or lung. Occurrence of metastatic disease is associated with a 5 years survival of >50% [1,2]. Currently, no methods for the identification, the prediction, or the cure of malignant pheochromocytomas are available. The development of malignancy and the underlying molecular pathways in PPGLs are poorly understood and efficient treatment strategies are missing.

**Figure 1:**
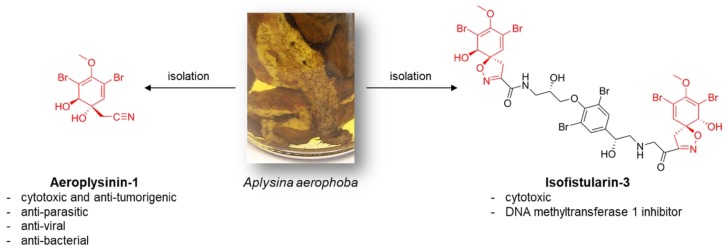
Schematic view: fresh collected 15 cm large *A. aerophoba* demosponge that grow under marine ranching conditions and the chemical structure of its bioactive secondary metabolites Aeropysinin-1 and Isofistularin-3.

Natural sources of potential anti-tumorigenic and anti-metastatic agents are, for example, provided by diverse marine sponges [3,4]. Lacking an effective external defense mechanism, marine sponges developed chemical defense strategies by the production of secondary metabolites with various bioactivities [5]. A prominent agent is the bioactive (+)-Aeroplysinin-1 isolated from the marine demosponge *Aplysina aerophoba* (Figure 1) [6], which is also the source of chitinous scaffolds for diverse biomedical [7,8] and technological [9] applications. Beside the antibiotic and antiviral activity, Aeroplysinin-1 triggers key molecules of the inflammatory response as especially cyclooxygenase-2 (COX-2), metalloproteinases 1 (MMP-1), and 2 (MMP-2) [10]. Moreover, Aeroplysinin-1 demonstrates an anti-angiogenic activity in vivo and in vitro [11]. An anti-tumorigenic effect of Aeroplysinin-1 has been demonstrated for two human breast cancer cell lines (ZR-75-1 and MCF-7). Sallam et al. [12] showed an inhibitory activity of several dibromotyrosine analogues of Aeroplysinin-1 on human prostate cancer proliferation, migration, and invasion. Treatment with Aeroplysinin-1 blocks the epidermal growth factor (EGF)-dependent proliferation probably due to the inhibition of EGF receptor phosphorylation [13]. Nevertheless, this receptor tyrosine kinase inhibitory activity of Aeroplysinin-1 was controversially discussed in the literature (reviewed in [11]). In conclusion, Aeroplysinin-1 addresses four hallmarks of cancer; proliferation, inflammation, angiogenesis, and metastasis, but the underlying mechanism is mainly unclear.

A less characterized brominated compound derived from *A. aerophoba* is Isofistularin-3 (Figure 1). Cytotoxic activity of Isofistularin-3 against HeLa cells has been reported (IC_50_ = 8.5 ± 0.2 µM) [14]. Recently, Florean et al. [15] described Isofistularin-3 as a new DNA methyltransferase (DNMT) 1 inhibitor. The agent reduces viability, colony formation as well as in vivo tumor growth in two lymphoma cell lines without affecting the viability of peripheral blood mononuclear cells or zebrafish development. The influence of Isofistularin-3 on cells´ pro-metastatic behavior has not yet been determined.

The absence of effective treatment strategies for malignant pheochromocytoma prompted us to investigate the anti-tumorigenic and anti-metastatic activity of Aeroplysinin-1 and Isofistularin-3 against pheochromocytoma cells in vitro. To the best of our knowledge, data on the impact of Aeroplysinin-1 and Isofistularin-3 pheochromocytoma cells are lacking. The choice to study bromotyrosines of the *A. aerophoba* demosponge origin was motivated by well-developed marine farming of this sponge species and its recognition as a renewable source to isolate large amounts of both bromotyrosines and chitin-based scaffolds [16].

## 2. Results

### 2.1. Anti-Proliferative Activity of Aeroplysinin-1 and Isofistularin-3 in Vitro

**Figure 2:**
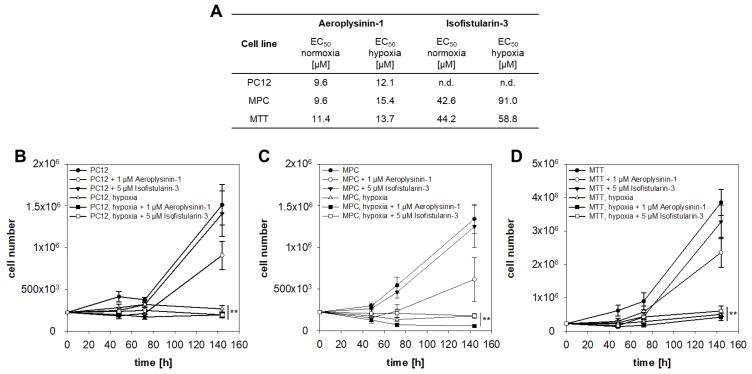
Anti-proliferative activity of Aeroplysinin-1 and Isofistularin-3 on pheochromocytoma cells in monolayer culture. (**A**) Aeroplysinin-1 decreased the viability of all three pheochromocytoma cell lines significantly after 24 h treatment. Isofistularin-3 only affected the viability of the mouse pheochromocytoma cells, whereas, the PC12 rat pheochromocytoma cells was not affected up to a concentration of 100 µM under normoxic and hypoxic conditions. Cultivation under hypoxia increased the necessary effective concentration to reduce the viability to 50% (EC_50_). (**B**) Furthermore, the effect on the number of proliferating cells was analyzed under normoxic and hypoxic conditions. Treatment with 1 µM Aeroplysinin-1 reduced the number of proliferating cells in all three cell lines in trend. Under hypoxic conditions (1% oxygen), pheochromocytoma cells stopped cell division. Four to five independent experiments were performed (*n* = 4–14). Average ± standard error of the mean (SEM); Analysis of variance (ANOVA) and Bonferroni *post hoc* test comparison vs. control * *p* < 0.05, ** *p* < 0.01.

To investigate the anti-proliferative activity of Aeroplysinin-1 and Isofistularin-3 against PPGLs, three different pheochromocytoma cell lines were used. Aeroplysinin-1 diminished the cell viability (Figure 2A and Figure S1) of all three cell lines in a micromolar concentration (EC_50_ = 10–11 µM). Twenty-four hours of incubation under extrinsic hypoxia (1% oxygen) in the presence of Aeroplysinin-1 resulted in a slight decrease of the effect (EC_50_ = 12–15 µM). Interestingly, 24 h incubation with Isofistularin-3 under normoxic or hypoxic conditions had no influence on the viability of the rat PC12 cells up to a concentration of 100 µM. The viability of mouse pheochromocytoma cell lines, MPC and MTT, was reduced in a high micromolar range (EC_50,normoxia_ = 43–44 µM; EC_50,hypoxia_ = 59–91 µM). Isofistularin-3 had no influence on the number of proliferating cells, whereas, Aeroplysinin-1 decreased the number of proliferating cells in all three cell lines (Figure 2B–D). The pheochromocytoma cell lines are affected by the reduction of oxygen and stopped cell division under hypoxic conditions.

**Figure 3:**
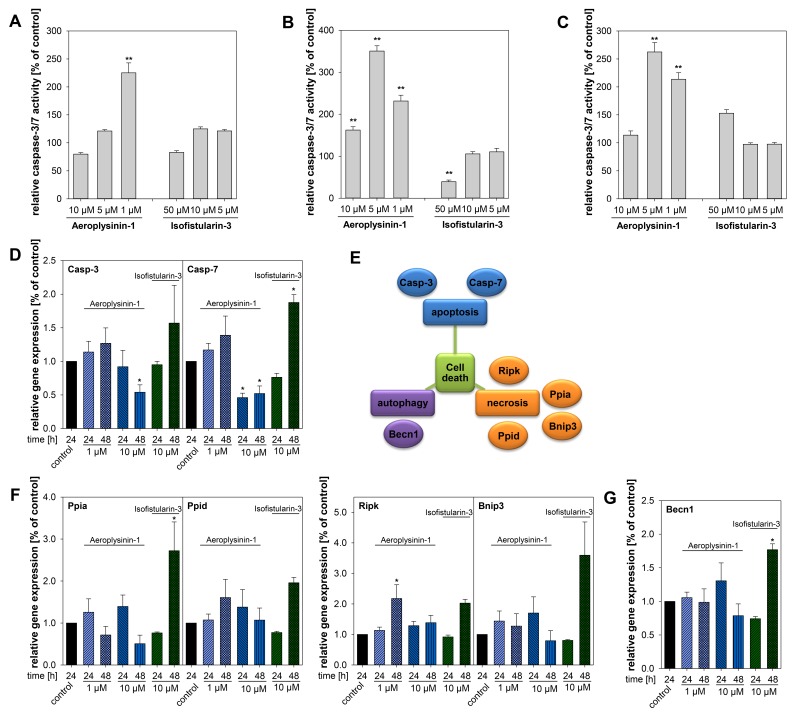
Influence of Aeroplysinin-1 and Isofistularin-3 on cell death related pathways. Induction of apoptosis by the treatment with Aeroplysinin-1 and Isofistularin-3 was analyzed by the measurement of the relative caspase-3 and caspase-7 activity in (**A**) MPC, (**B**) MTT, and (**C**) PC12 cells. Moreover, the impact on the (**D**–**G**) gene expression levels of the MTT cells was determined by qRT-PCR. (**E**) Beside apoptosis, necrosis and autophagy are also forms of cell death regulated by several genes. (**F**) The analyzed markers for necrosis *Ppia* (cyclophilin A), *Ppid* (cyclophilin D), *Ripk* and *Bnip3* were not affected by the treatment with Aeroplysinin-1. (**G**) Furthermore, no difference of the *Becn1* expression level was detectable after Aeroplysinin-1 treatment. Three to four independent experiments (*n* = 3–4). Average ± SEM; ANOVA and Bonferroni *post hoc* test comparison vs. control * *p* < 0.05 or ** *p* < 0.01.

Aeroplysinin-1 induced apoptosis, analyzed by caspase (casp)-3 and casp-7 activity assay, in a concentration-dependent manner but predominant at lower concentrations (Figure 3A–C). Furthermore, gene expression analysis demonstrated a reduction of *casp-3* and *casp-7* after treatment with 10 µM Aeroplysinin-1 in the MTT cells (Figure 3D). In contrast to the programmed cell death during apoptosis, necrosis causes traumatic cell death due to an acute cellular injury. Several markers for necrosis (e.g., cyclophilin A and D, RIPK, and BNIP3) were described, but gene expression analysis showed no difference in the expression of these genes after treatment with Aeroplysinin-1 (Figure 3E,F). Moreover, Belcin 1 (*Becn1*) a marker of cellular stress and a crucial regulator of the crosstalk between apoptosis and autophagy [17], was not affected by the treatment with Aeroplysinin-1 (Figure 3G).

Isofistularin-3 reduced caspase activity at higher concentrations (50 µM) (Figure 3A–C), whereas, expression of the caspase genes was induced after 48 h treatment with a concentration of 10 µM. The necrosis-associated genes (Figure 3E,F) were up-regulated after 48 h treatment with Isofistularin-3. Furthermore, *Becn1* as a marker of autophagy was increased after Isofistularin-3 treatment (Figure 3G).

**Figure 4:**
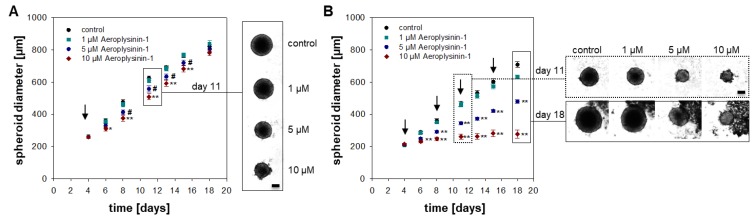
Spheroid growth inhibition by a single or fractionated treatment with Aeroplysinin-1. (**A**) Spheroids were treated with different concentrations Aeroplysinin-1 after completed spheroid formation (day 4) and growth was monitored over a time period of 14 days. Furthermore, (**B**) a fractionated treatment on day four, 8, 11, and 15 took place. Three to four independent experiments (*n* = 15–48). Average ± SEM; ANOVA and Bonferroni *post hoc* test comparison vs. control * *p* < 0.05, ** *p* < 0.01; or vs. 5 µM Aeropylysinin-1 # *p* < 0.05. Arrows mark the different treatment time points. Scale bar: 200 µm.

Our investigations in monolayer culture demonstrate the promising effects of Aeroplysinin-1 as an anti-proliferative agent. In the next step, we aimed to determine whether Aeroplysinin-1 is also effective in a more complex model that is closer to the in vivo conditions by the formation of therapeutic resistant hypoxic and necrotic areas. Therefore, 3-dimensional tumor cell spheroids of the MTT cells were generated. Tumor cell spheroids are characterized by a pH, oxygen, and nutrient gradient and allow the screening of different drugs with regard to their potential anti-tumorigenic activity [18]. A single treatment with 5–10 µM Aeroplysinin-1 diminished the spheroid growth significantly over a time period of 12 days (Figure 4A). The medium of the spheroids was replaced every four days. Consequently, four days after treatment there was no Aeroplysinin-1 remaining in the medium. A single treatment with Aeroplysinin-1 resulted in a decelerated spheroid growth, but was not able to inhibit spheroid growth completely. Therefore, we decided to perform a fractionated treatment as usually performed in the clinic. Treatment with Aeroplysinin-1 took place on day four, 8, 11, and 15 after spheroid generation and resulted in a full inhibition of spheroid growth at a concentration of 10 µM (Figure 4B). Moreover, even 5 µM Aeroplysinin-1 significantly decreased spheroid growth over the entire time period. To sum up, Aeroplysinin-1 showed promising anti-proliferative activity in monolayer as well as spheroid culture of pheochromocytoma cells, indicating a potential anti-tumorigenic activity of this sponge-derived drug. 

### 2.2. Influence of Aeroplysinin-1 and Isofistularin-3 on Cells’ Pro-Metastatic Behavior

**Figure 5:**
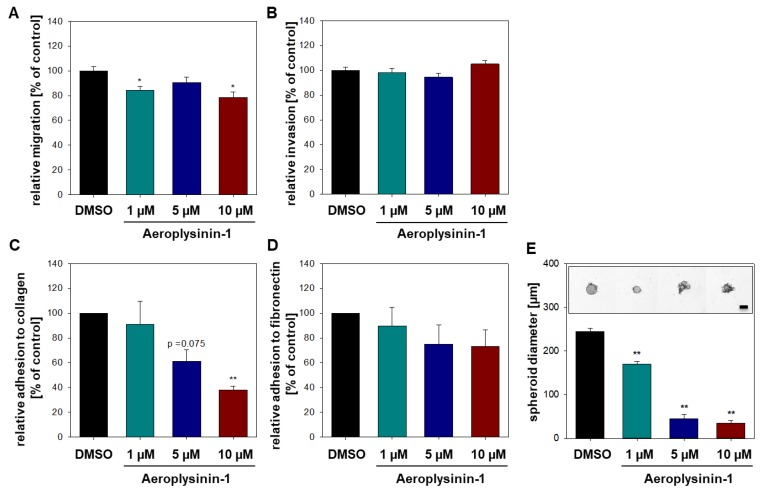
Aeroplysinin-1 influences the pro-metastatic behavior of MTT cells. Impact of different concentrations of Aeroplysinin-1 on (**A**) MTT cell migration and (**B**) invasion were analyzed in Boyden-Chamber assays (with (**B**) or without (**A**) Matrigel coating) after 24 h. Furthermore, the adhesion capacity of MTT cells to (**C**) collagen and (**D**) fibronectin after 24 h treatment with Aeroplysinin-1 was determined. (**E**) Spheroid formation was tracked over four days and the spheroid diameter was analyzed four days after seeding. Three independent experiments (*n* = 12–18). Average ± SEM; ANOVA and Bonferroni *post hoc* test comparison vs. control * *p* < 0.05 or ** *p* < 0.01. Scale bar: 200 µm.

Beside the inhibition of tumor growth, the prevention of the development of tumor metastases is a major goal of a potential successful cancer treatment. Formation of metastases is particularly dependent on the migration, invasion, and adhesion capacity of a single cell (pro-metastatic properties). In this context, we asked whether the sponge-derived marine drugs Aeroplysinin-1 and Isofistularin-3 are able to reduce the pro-metastatic behavior of pheochromocytoma cells. To determine the effect of Aeroplysinin-1 and Isofistularin-3 on the migration and invasion capacity of MTT cells Boyden-Chamber assays with or without Matrigel^®^ coating were performed. Aeroplysinin-1 decreased the migration ability of the cells significantly, whereas, the invasion capacity was not affected (Figure 5A,B). In contrast, Isofistularin-3 had no influence on MTT cell migration and invasion (Figure S2). Furthermore, the adhesion to the extracellular matrix proteins collagen and fibronectin was analyzed (Figure 5C,D). MTT cells adhered with a comparable low affinity to fibronectin that was mildly affected by treatment with Aeroplysinin-1. In contrast, Aeroplysinin-1 significantly diminished the high adhesion capacity of the MTT cells to collagen. The adhesion capacity of the pheochromocytoma cell was not affected by Isofistularin-3 (Figure S2). To further investigate the ability of Aeroplysinin-1 to prevent the formation of metastases the effect of this compound on spheroid formation was examined. Therefore, cell suspension was treated with different concentrations of Aeroplysinin-1 before cells were seeded to form spheroids. A concentration of 1 µM Aeroplysinin-1 significantly inhibits the ability of MTT cells to form spheroids (Figure 5E). These data indicate a potential influence of Aeroplysinin-1 on cell adhesion molecules (e.g., integrins, cadherins, and selectins) and, moreover, gives a first hint regarding the potential anti-metastatic effects of this sponge-derived drug.

### 2.3. Impact of Aeroplysinin-1 on Cell Adhesion Molecules

**Figure 6:**
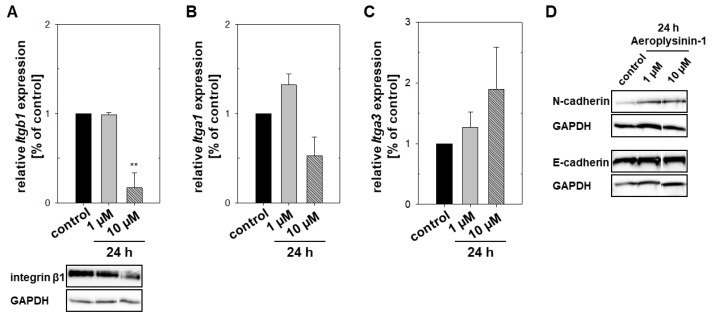
Regulation of cell adhesion molecules in mouse pheochromocytoma cells (MTT) by Aeroplysinin-1. The impact of 24 h treatment with Aeroplysinin-1 on the gene expression was confirmed by qRT-PCR and the protein expression was analyzed by western blot analysis. Three to four independent experiments were performed (*n* = 3–4) and a representative section of the immunochemical detection is shown. Treatment with 10 µM Aeroplysinin-1 diminished the expression of integrin β1 (**A**) significantly, whereas, the gene expression of *Itga1* (**B**) and *Itga3* (**C**) was not affected. Furthermore, Aeroplysinin-1 had no impact on the protein expression of the calcium-dependent cadherins. Average ± SEM; ANOVA and Bonferroni *post hoc* test comparison vs. control ** *p* < 0.01.

Cell adhesion molecules (CAMs) are the major players for the binding with other cells and the extracellular matrix (ECM). In consideration with the previous results, we hypothesized that Aeroplysinin-1 influences the expression of these CAMs. Integrins are responsible for the cell interaction with the ECM glycoproteins (e.g., collagen, fibronectin, and laminin) and are heterodimeric proteins consisting of alpha and beta subunits. Here we focused on the integrins α1β1 (ligands: collagens, laminins), α2β1 (ligands: collagens, laminins), and α4β1 (ligands: fibronectin, VCAM-1). An up-regulation of ITGB1 is associated with a pro-metastatic behavior, for example in lung [19] and breast cancer [20], as well as renal cell carcinomas [21]. A concentration of 10 µM Aeroplysinin-1 repressed the expression of the beta subunit (Itgb1) in the MTT cells significantly, whereas the expression of *Itga1* and *Itga3* was not affected by Aeroplysinin-1 (Figure 6A–C). MTT cells showed no *Itga4* expression that could explain the relatively low affinity of MTT cells to fibronectin.

A second type of CAMs is the calcium-dependent cadherins. These transmembrane proteins are important for the formation of adherens junctions, which are crucial for cell-cell interactions. A loss of epithelial(E)-cadherin (CDH1) expression or function diminishes the strength of cellular adhesion within a tissue and results in an increase in cellular motility that correlates with tumor progression and metastasis [22]. Furthermore, the expression of neuronal(N)-cadherin (CDH2), normally expressed from mesenchymal cells, promotes cellular motility and the invasiveness of tumor cells. This switch from E-cadherin to N-cadherin has a functional significance in cancer metastasis [23]. Treatment with Aeroplysinin-1 had no impact on the protein expression of E- and N-cadherin (Figure 6D). Beta-catenin as a subunit of the cadherin protein complex is also involved in the regulation and coordination of cell-cell adhesion and acts as a signal transducer in the Wnt signaling pathway. Park et al. [24] demonstrated that Aeroplysinin-1 inhibits the proliferation of colon cancer cells by promoting β-catenin degradation. Beta-catenin gene expression was significantly reduced after 48 h treatment with Aeroplysinin-1 (data not shown) indicating an impact of Aeroplysinin-1 on the Wnt/β-catenin signaling.

## 3. Discussion

For the cure of metastatic PPGLs effective treatment strategies are missing. In our present study, we evaluate the impact of two secondary metabolites, Aeroplysinin-1 and Isofistularin-3, isolated from the marine demosponge *A. aerophoba* on the proliferative and pro-metastatic behavior of pheochromocytoma cell lines.

Aeroplysinin-1 diminished the number of proliferating pheochromocytoma cells and their viability in a micromolare range. Furthermore, the MTT cell spheroid growth was significantly repressed by the fractionated treatment with Aeroplysinin-1. The induction of apoptosis only occurred at a lower dose (1–5 µM) and the hypothesis that the effects of higher concentrations are related to necrosis or autophagy could not be confirmed. The impact on cell viability is in line with the findings of other groups on different tumor cell lines (reviewed in [11]). Koulman and coworkers concluded that the formation of free radicals by the semiquinone structure is at least contributing to the cytotoxicity of Aeroplysinin-1 [25]. Another sign in this direction is provided by the work from Stuhldreier et al., who demonstrate that Aeroplysinin-1 stimulates the phosphorylation of histone H2AX (γ-H2AX), a marker for DNA damage, in acute myeloid (NOMO-1) and acute monocytic (THP-1) cells [26]. Moreover, an EGF-dependent anti-proliferative effect was described by the group of Kreuter [13]. The inhibition of receptor tyrosine kinases that are involved in the transduction of mitogenic signals could furthermore be responsible for the effect of Aeroplysinin-1 on cell growth and proliferation [27]. Our data provide a first hint that Aeroplysinin-1 could demonstrate an anti-tumorigenic activity on PPGLs, however, additional in vivo experiments are needed to confirm these findings. Moreover, a specific therapeutic target of Aeroplysinin-1 is largely unknown and that is why the toxicity to the normal tissue must be evaluated carefully to identify unintentional negative side-effects. Anti-angiogenic activity [28,29] and the previously presented data underline the potential anti-tumorigenic activity of Aeroplysinin-1 on the one hand, but indicate an impact on normal blood vessel endothelial cells on the other hand. Kreuter and colleagues demonstrate that Aeroplysinin-1 blocked the proliferation of two breast cancer cell lines (0.25–0.5 µM) significantly; whereas a 10-fold higher concentration did not reveal any cytotoxicity on human fibroblast [13,30].

Cancer cell metastasis is a multistep cascade that is dependent on dynamic changes in the adhesive and migratory ability of tumor cells. Aeroplysinin-1 decreased the migration ability of pheochromocytoma cells significantly, whereas the invasion capacity was not affected. Furthermore, treatment with Aeroplysinin-1 reduced the adhesion capacity of the MTT cells. Quantitative RT-PCR and western blotting confirmed a reduction of the integrin β1 expression after 24 h treatment. The integrin αvβ3 and αvβ5 antagonist cilengitide showed favorable results in clinical phase 2 trials in patients with glioblastoma [31,32], non-small-cell lung [33,34], and prostate [35] cancer (reviewed [36]). Therapeutic strategies targeting integrin β1 have also shown efficacy to reduce tumor growth in preclinical models. Volociximab, a monoclonal antibody blocking the function of integrin α5β1 inhibits angiogenesis and decreases tumor growth in vitro [37]. A phase 1 trial in patients with advanced solid malignancies [38] and a phase 2 trial in patients with platinum-resistant advanced epithelial ovarian or primary peritoneal cancer [39] demonstrated that Voleciximab was well tolerated and may improve the clinical outcome. A peptide antagonist of integrin α5β1 with anti-angiogenic activity, ATN-161, prolonged stable disease in one-third of the patients with advanced solid tumors [40]. Interestingly, Motuporamine C, a cytotoxic alkaloid isolated from the marine sponge *Xestospongia exigua*, inhibits the invasion of breast and prostate carcinoma and glioma cell lines by a reduction of the integrin ß1 activity [41]. ß1 integrins induce adhesion-dependent activation of the focal adhesion kinase (FAK) and proto-oncogene tyrosine-protein kinase SRC, leading to proliferation, migration, invasion and the survival of tumor cells bound to the ECM [36]. Consequently, a downregulation of β1 integrin via Aeroplysinin-1 offers an excellent therapeutic opportunity to suppress tumor cell migration and invasion, and could furthermore explain the anti-proliferative activity of this sponge-derived secondary metabolite. Further studies in vivo are necessary to confirm our promising in vitro results.

An increased resistance of several tumors against commonly used treatment modalities, such as radiation therapy and chemotherapy, is a major limitation in the therapeutic regime of these tumors. The tumor microenvironment and extracellular matrix play a critical role in the response to different therapeutic options. β1 integrin is described as promising molecular target to enhance radiation therapy [42] and modulate the chemotherapy resistance [43]. A combination of Aeroplysinin-1 with one of the common treatment modalities should be the objective of further studies to evaluate the potential of Aeroplysinin-1 as potential chemo-/radio-sensitizing agent. This could be important especially for PPGLs with an activation in pseudohypoxic pathways, including those with mutations in HIF2α, VHL, PHD and particularly SDHB that carry a higher risk of malignancy [44]. Moreover, stabilization of hypoxia-inducible factors (HIFs) promotes the expression of integrin β1 [45].

In contrast to the auspicious impact of Aeroplysinin-1 on the proliferative and pro-metastatic behavior of the pheochromocytoma cell lines, Isofistularin-3 showed only a negligible activity on these cell properties. In line with the findings from Florean and coworkers, our qRT-PCR results indicate an induction of autophagy- and necrosis-related genes after 48 h treatment [15]. Nevertheless, an impact on the number of proliferating cells was not detected in our pheochromocytoma cell model.

For the first time, our in vitro investigations show promise for the application of Aeroplysinin-1 as anti-tumorigenic and anti-metastatic agent against PPGLs. We suggest that the mechanism is based on the reduction of the integrin β1 expression. The application of Aeroplysinin-1 and other sponge-derived secondary metabolites provides a promising therapeutic strategy especially for the treatment of metastatic PPGLs and other tumor entities with the tendency for metastatic spread.

## 4. Materials and Methods

BromMarin GmbH (Freiberg, Germany) kindly provided Isofistularin-3 and Aeroplysinin-1 with the purity grade of 99.9%.

### 4.1. Cell Culture

The mouse pheochromocytoma cells (MPC) generated from heterozygous neurofibromatosis knockout mice and its more aggressive derivate termed MTT as well as the rat pheochromocytoma cell line PC12 were acquired from Arthur Tischler [46,47,48]. For the cultivation of the PC12 cells RPMI-1640 containing 10% horse serum (HS) and 5% fetal calf serum (FCS) were used. The additional supplementation of this medium with 2 mM Glutamax is necessary for the MPC cell cultivation. The MTT cells were cultivated in Dulbecco’s Modified Eagle Medium (DMEM) plus Glutamax supplemented with 10% HS, 5% FCS, and 1 mM sodium pyruvate. In general, cells were cultivated under normoxic conditions in a CO_2_ incubator. In order to simulate hypoxic conditions (extrinsic hypoxia) cells were cultivated at reduced oxygen partial pressure (≤1% O_2_) in a special incubator equipped with an oxygen-sensor (Gasboy, Labotect, Göttingen, Germany). In all cases, cells were cultured at 37 °C, 5% CO_2_, and 95% humidity. MycoAlert Mycoplasma Detection Kit (Lonza, Basel, Switzerland) was used for testing cells to be mycoplasma free. After trypsinization (trypsin/EDTA; 0.05%/0.02%) cells were diluted with complete medium (DMEM plus Glutamax with 10% HS and 5% FCS) and counted by using C-CHIPs (Neubauer improved). All experiments were performed antibiotic free after at least one passage after re-cultivation. The cultivation and the experimental work were performed by using collagen A coated cell culture dishes.

### 4.2. Viability Assay

To investigate the effect of Isofistularin-3 and Aeroplysinin-1 on cell viability the CellTiter 96^®^ AQueous One Solution Cell Proliferation Assay (Promega, Mannheim, Germany) was used. In analogy to manufacturer´s instructions both cell lines (1.75 × 10^4^) were seeded in 96-well plates and incubated for 24 h with different concentrations of the compounds. After 3 h incubation at 37 °C with CellTiter 96^®^ AQueous One reagent the absorption of the whole plate was measured at 490 nm by Anthos htIII plate reader. The half maximal effective concentration (EC_50_) was calculated from the dose-response curve by using the dose-response fit model of the SigmaPlot 12.5 software package (SYSTAT Software, San Jose, CA, USA).

### 4.3. Proliferation Assay

2.25 × 10^5^ cells were seeded in 6-well plates and allowed to attach for 24 h. Cells were treated with Isofistularin-3 or Aeroplysinin-1 (1 µM or 10 µM) and cultivated for 48 h, 72 h, or 144 h under normoxic or hypoxic conditions. After incubation, cells were washed with PBS, trypsinated, and after careful resuspension in medium (total volume: 1 mL) cells were counted by using C-CHIPs (Neubauer improved). Each well was counted in duplicate.

### 4.4. Apoptosis Assay

To determine the effect of Isofistularin-3 and Aeroplysinin-1 on cell viability the Apo-ONE^®^ Homogeneous Caspase-3/7 Assay (Promega, Germany) was used. In analogy to the manufacturer´s instructions all cell lines (1.75 × 10^4^) were seeded in 96-well plates. Cells were treated with different concentrations of Isofistularin-3 and Aeroplysinin-1 for 24 h under normoxic or hypoxic conditions. After 1 h incubation at room temperature with 80 µL of the supernatant was transferred (duplicate) in a black 96-well plate and the fluorescence was analyzed at 485_Ex_/530_Em_ by LB940 Multilabel Reader Mithras (Berthold Technologies GmbH Co. KG, Bad Wildbad, Germany; Software: MikroWin 2000, Labsis Laborsysteme GmbH, Neunkirchen-Seelscheid, Germany).

### 4.5. Migration Assay

The influence of Aeroplysinin-1 and Isofistularin-3 on the ability of MTT cells to migrate through 8 µm pores was ascertained by using TC-Inserts (Item No. 83.3931.800; Sarstedt AG & Co. KG, Nümbrecht, Germany). 5 × 10^6^ cells were plated in a cell culture flask (T75) and cultivated for 24 h under normoxia. Medium was removed and DMEM plus Glutmax containing 0.2% bovine serum albumin (BSA) was added, followed by 24 h incubation. Cells were washed with PBS, trypsinized, and different concentrations of Aeroplysinin-1 (10, 5 and 1 µM), or Isofistularin-3 (10 and 5 µM), or DMSO as control was added to the cell suspension containing 1 × 10^6^ cells per milliliter in DMEM containing 0.2% BSA. As chemoattractant complete DMEM + Glutamax (10% HS, 5% FCS, 1 mM sodium pyruvate) was filled in each well of a 12-well plate and the treated single cell suspensions (2 × 10^5^ cells/insert) were added in the upper compartment of the cell culture insert. After 24 h incubation, culture medium was replaced by DMEM + Glutamax (0.2% BSA) with 1 µM calcein (BD^TM^ calcein AM Fluorescent Dye, BD Biosciences, Franklin Lakes, NJ, USA) for 1 h at 37 °C. Afterwards, the lower compartment was washed with PBS and the cells that migrated through the pores were trypsinized and the fluorescence of the calcein-stained cells was measured at 485_Ex_/528_Em_ by VICTOR3 1420 Multilabel Counter (Perkin Elmer, Hong Kong, China).

### 4.6. Invasion Assay

For invasion experiments the TC-Inserts were coated with basement membrane matrigel (Matrigel (BD Bioscience)/DMEM + Glutamax, 1/3, *v*/*v*) and the experimental procedure was analog to the migration assay above.

### 4.7. Adhesion Assay

4 × 10^5^ cells were plated in each well of a 6-well plate (pre-culture). After 24 h cells were treated with different concentrations of Aeroplysinin-1 or Isofistularin-3. DMSO was used as a control. Furthermore, 24-well plates were coated with human fibronectin (5 mg/mL in PBS, Biochrom Ltd., Cambridge, UK) overnight at 4 °C or as previously described with collagen A. After 24 h the fibronectin or collagen coated plates were washed two times with PBS and unspecific binding sites were blocked with PBS containing 2% BSA for 1 h at 37 °C. The 24 h treated cells were washed two times with PBS, detached with trypsin (comment: detachment with EDTA or accutase was not useful for the cells) and resuspended in DMEM + Glutamax containing 0.2% BSA. 2 × 10^5^ cells per well that were seeded in the fibronectin- or collagen-coated wells and allowed to adhere for 30 min. Non-adherent cells were washed away with PBS. The remaining cells were fixed for 5 min in PBS/methanol followed by 10 min in 100% methanol and stained for 15 min with crystal violet. After four washing steps (tap water) cells were dried on air and lysed by using PBS containing 0.5% Triton-X-100 for 30 min under continuous shaking. After transfer in a 96-well plate, absorption at 550 nm (reference 650 nm) was measured by VICTOR3 1420 Multilabel Counter. PBS containing 0.5% Triton-X-100 was used as blank.

### 4.8. Generation and Cultivation of Tumor Cell Spheroids

5 × 10^3^ cells resupended in complete cell culture medium containing 20% of a 1.2% methylcellulose solution (0.24% (*w*/*v*), prepared in DMEM + Glutamax) were seeded in nonadherent round-bottom 96-well plates for suspension culture (Greiner, Kremsmünster, Austria). After 3–4 days´ cultivation consumed medium was replaced. To determine the influence of Aeroplysinin-1 or Isofistularin-3 four days’ old spheroids were treated with different concentrations of the marine drugs. The treatment of the spheroids takes place one-time and was removed after 4 days by the medium replacement (Figure 4A). In a second experimental setting a fractionated treatment of spheroids was performed to determine if a fractionated treatment is able to reduce the necessary dose (Figure 4B). Therefore, spheroids were treated at day 4, 8, 11, and 15. The size of each spheroid was measured by using an inverse microscope Axiovert 200 M (Software: AxioVision 4.8; Carl Zeiss AG, Oberkoch, Germany). The area (A) of each spheroid was analyzed by using the software package Fiji (ImageJ, 1.51t, National Institutes of Health, Bethesda, MD, USA). The diameter (d) was calculated under acceptance of an approximately spherical form of the spheroids (d = 2 × √(A/π)).

### 4.9. Tumor Cell Spheroid Formation Assay

To analyze the influence of Aeroplysinin-1 or Isofistularin-3 on the formation of the MTT cell spheroids the cell suspension containing 20% of a 1.2% methylcellulose solution was treated with different concentrations of the marine drug. After seeding, formation was tracked over a time period of four days.

### 4.10. RNA Isolation

RNA from cell pellets was isolated using RNeasy Plus Mini kit (Qiagen, Hilden, Germany) according to the manufacturer’s instructions. RNA concentration was analyzed using BopPhotometer (Eppendorf, Hamburg, Germany). For the reverse transcription of 1 µg RNA the iScript RT kit (Bio-Rad, Hercules, CA, USA) was utilized. One microliter of the transcribed cDNA per reaction was used for qRT-PCR analysis.

### 4.11. Quantitative Real-Time PCR

Mouse *ß-actin*, *Ripk*, *Bnip3*, *Ppia*, *Ppid*, *Becn1*, *Casp3*, *Casp7*, *Cdh1*, *Cdh2*, *Itga1*, *Itga3*, *Itga4*, *Itgb1* mRNA expression was analyzed using the Quantitec SYBR PCR Master Mix (Qiagen GmbH, Hilden, Germany) and was expressed relative to ß-actin RNA levels as internal control. The sequence of each primer pair and the optimized annealing temperature is described in detail in the supporting information (Table S1). The amplification protocol consisted of a denaturation step at 95 °C for 7 min, followed by 40–45 cycles with a 95 °C denaturation step for 15 s, an annealing step for 20 s, and the extension step at 72 °C for 15 s. The expression of all genes was determined using Bio-Rad CFX 384 Real-Time System (Bio-Rad) and was analyzed using the comparative threshold cycle (CT) method [49].

### 4.12. SDS-PAGE and Western Blotting

Protein synthesis of integrin β1, E-cadherin, N-cadherin, and GAPDH was determined by Western blot analysis. After incubation with Aeroplysinin-1 under normoxic or hypoxic conditions, cells were washed with PBS, detached with trypsin/EDTA, and resuspended in cold medium. After centrifugation at 4 °C the obtained pellet was washed twice with PBS and stored at −80 °C. Whole cell lysates were prepared on ice using CellLytic^TM^ M (Sigma-Aldrich, St. Louis, MO, USA, C2978) with protease inhibitors (1:100, Sigma-Aldrich; P8340). After 30 min incubation and thoroughly mixing, cell lysate were centrifuged to remove cell debris. The protein concentration of each supernatant was quantified using Bradford assay. Thirty microgram proteins were mixed with four-fold LDS sample buffer (C.B.S. Scientific, San Diego, CA, USA; FB31010) and 5% mercaptoenthanol, denatured at 99 °C for 5 min and separated by 10% SDS-polyacrylamide gels (PAGE). Protein transfer to a polyvinylidine difluoride membrane (0.45 µm; Whatman, Maidstone, UK) was performed by semi-dry electroblotting. Non-specific binding sites on the membrane were blocked by one hour incubation with 5% skimmed milk powder plus 2% bovine serum albumin in TBS-T (blocking solution) at room temperature. Membrane was incubated with primary antibodies anti-integrin beta 1 (1:500; ab179471; abcam plc., Cambridge, UK), anti-E cadherin (1:500, ab76319; abcam plc.), anti-N cadherin (1:500; ab18203; abcam plc.), and anti-GAPDH (1:1000; #2118, Cell Signaling Technology, Frankfurt am Main, Germany) for one hour at room temperature followed by an overnight incubation at 4 °C. After three washing steps in TBS-T, membranes were incubated for one hour at room temperature with peroxidase-conjugated secondary antibody goat anti-rabbit IgG (1:5000; sc-2004; Santa Cruz Biotechnology, Dallas, TX, USA) or goat anti-mouse IgG (1:5000; sc-2005; Santa Cruz Biotechnology). All antibodies were diluted in blocking solution. After washing with TBS-T, proteins were visualized by chemiluminescence under the use of SuperSignal^®^ West Pico and Femto Chemiluminescent Substrate (Thermo Fisher Scientific, Waltham, MA, USA) and detected using G:BOX Chemi-XL1.4 imaging system from Syngene (Cambridge, UK).

### 4.13. Statistical Analysis

Descriptive data were expressed as average ± SEM. The number of n represents the number of technical and biological replicates within the independent experiments. Statistical analyses were carried out by a one-way analysis of variance with post hoc Bonferroni using SigmaPlot 12.5 (Systat Software GmbH, Erkrath, Germany).

## Supplementary Materials

The following are available online at http://www.mdpi.com/1660-3397/16/5/172/s1, Table S1: Primer sequences and the targeted genes, Figure S1: Dose-response curves of three different pheochromocytoma cell lines after treatment with Aeroplysinin-1 or Isofistularin-3 under normoxic and hypoxic conditions, Figure S2: Impact of Isofistularin-3 on the pro-metastatic behavior of MTT cells.
