# Supplementary material for: Anti-Tumorigenic and Anti-Metastatic Activity of the Sponge-Derived Marine Drugs Aeroplysinin-1 and Isofistularin-3 against Pheochromocytoma In Vitro
Source: Mar Drugs. 2018 May 20;16(5):172. doi: 10.3390/md16050172 (PMC5983303; doi:10.3390/md16050172)
Supplement: Supplementary file 1 [file marinedrugs-16-00172-s001.pdf]

Supplementary material

Anti-tumorigenic and anti-metastatic activity of the sponge-derived marine drugs Aeroplysinin-1 and Isofistularin-3 against pheochromocytoma *in vitro*

Nicole Bechmann [1,*], Hermann Ehrlich [2], Graeme Eisenhofer [1,3], Andre Ehrlich [4], Stephan Meschke [4], Christian G. Ziegler [3], and Stefan R. Bornstein [3,5]

[1] Institute of Clinical Chemistry and Laboratory Medicine, University Hospital Carl Gustav Carus, Technical University Dresden, Fetscherstrasse 74, Dresden, Germany; Nicole.bechmann@uniklinikum-dresden.de; Graeme.eisenhofer@uniklinikum-dresden.de;

[2] Institute of Experimental Physics, TU Bergakademie Freiberg, Leipziger 23, 09599 Freiberg, Germany; Hermann.Ehrlich@physik.tu-freiberg.de

[3] Department of Medicine III, University Hospital Carl Gustav Carus, Technical University Dresden, Fetscherstrasse 74, 01307 Dresden, Germany; Stefan.bornstein@uniklinikum-dresden.de; Christian.ziegler@uniklinikum-dresden.de

[4] BromMarin GmbH, Wernerstraße 1, 09599 Freiberg; andre.ehrlich@brommarin.de; Stephan.meschke@brommarin.de

[5] Center for Regenerative Therapies Dresden, Technical University Dresden, Fetscherstrasse 105, 01307 Dresden, Germany

***** Correspondence: Nicole.bechmann@uniklinikum-dresden.de; Tel.: +49-351-458-19687

Contents

1. Experimental information's: qPCR 2
2. Influence of Aeroplysinin-1 and Isofistularin-3 on cell viability 2
3. Impact of Isofistularin-3 on cells´ pro-metastatic behavior 4

*Corresponding author: Institute of Clinical Chemistry and Laboratory Medicine, Technical University Dresden, Fetscherstrasse 74, Dresden, Germany; *Tel*: +49 351 45819687; *E-mail address*: Nicole.bechmann@uniklinikum-dresden.de

1. Experimental information's: qPCR

Table 1: Primer sequences and the targeted genes

Gene	Forward primer sequence	Reverse primer sequence	bp
mouse *ß-actin*	GAGCACAGCTTCTTTGCAGCTCCTT	TGCCATGTTCAATGGGGTACTTCAG	280
mouse *Ripk*	GAAGACAGACCTAGACAGCGG	CCAGTAGCTTCACCACTCGAC	182
mouse *Bnip3*	TCCTGGGTAGAACTGCACTTC	GCTGGGCATCCAACAGTATTT	103
mouse *Ppia*	GAGCTGTTTGCAGACAAAGTTC	CCCTGGCACATGAATCCTGG	125
mouse *Ppid*	AACCCGCGAGTCTTCTTTGAC	TAATTCGGTGGAAAGGGCATC	187
mouse *Becn1*	ATGGAGGGGTCTAAGGCGTC	ATGGAGGGGTCTAAGGCGTC	197
mouse *Casp3*	TGGTGATGAAGGGGTCATTTATG	TTCGGCTTTCCAGTCAGACTC	105
mouse *Casp7*	GGACCGAGTGCCCACTTATC	TCGCTTTGTCGAAGTTCTTGTT	89
mouse *Itga1* (Integrin alpha 1)	CCTTCCCTCGGATGTGAGTCA	AAGTTCTCCCCGTATGGTAAGA	106
mouse *Itga3* (Integrin alpha 3)	CCTCTTCGGCTACTCGGTC	CCGGTTGGTATAGTCATCACCC	112
mouse *Itga4* (integrin alpha 4)	GATGCTGTTGTTGTACTTCGGG	ACCACTGAGGCATTAGAGAGC	189
mouse *Itgb1* (integrin beta 1)	ATGCCAAATCTTGCGGAGAAT	TTTGCTGCGATTGGTGACATT	209

2. Influence of Aeroplysinin-1 and Isofistularin-3 on cell viability

Additionally to the calculated EC_{50} values in figure 2 the dose-response up to a concentration of 50 µM of Aeroplysinin-1 or 100 µM Isofistularin-3 are shown in Figure S1.

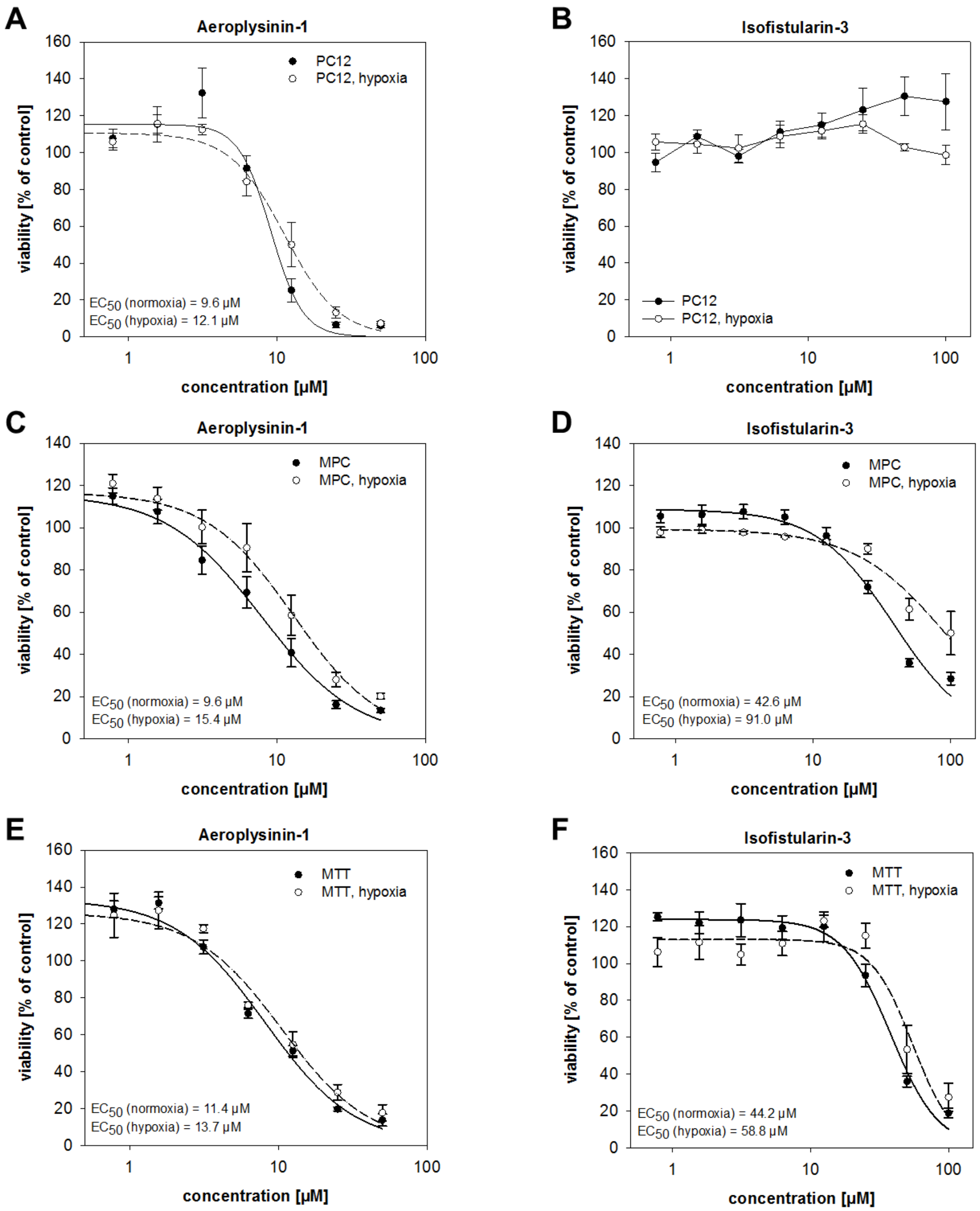

Figure S1: Dose-response curves of three different pheochromocytoma cell lines after treatment with Aeroplysinin-1 or Isofistularin-3 under normoxic and hypoxic conditions.

The effect of Aeroplysinin-1 (**A**, **C**, **E**) and Isofistularin-3 (**B**, **D**, **F**) on the viability of PC12 (**A**-**B**), MPC (**C**-**D**), and MTT (**E**-**F**) cells was analyzed by determining the dose-response curves after 24 h treatment using CellTiter 96® AQueous One Solution Cell Proliferation Assay. Half-maximal effective concentration (EC_{50}) was calculated from dose-response curve by using dose-response fit model. Four independent experiments (n = 4). Average±SEM.

3. Impact of Isofistularin-3 on cells´ pro-metastatic behavior

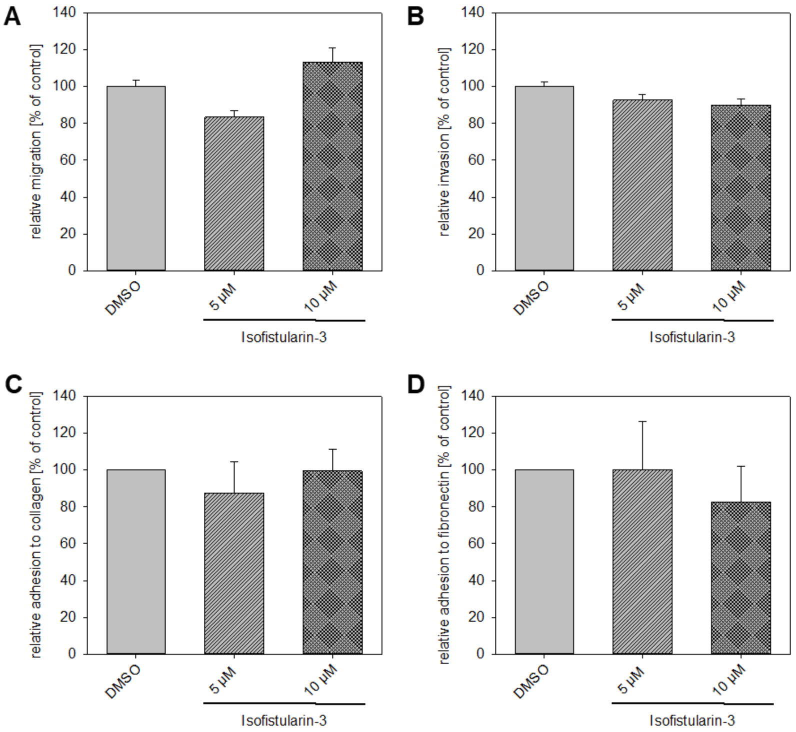

Figure S2: Impact of Isofistularin-3 on the pro-metastatic behavior of MTT cells.

Influence of different concentrations of Isofistularin-3 on (**A**) MTT cell migration and (**B**) invasion were analyzed in Boyden Chamber assays (with (**B**) or without (**A**) matrigel coating) after 24 h. Furthermore, the adhesion capacity of MTT cells to (**C**) collagen and (**D**) fibronectin after 24 h treatment with Aeroplysinin-1 was determined. Three to four independent experiments (n=12-18). Average±SEM; ANOVA and Bonferroni post *hoc* test comparison *vs.* control *p<0.05.
